# Supplementary material for: ProteinShader: illustrative rendering of macromolecules
Source: BMC Struct Biol. 2009 Mar 30;9:19. doi: 10.1186/1472-6807-9-19 (PMC2672931; doi:10.1186/1472-6807-9-19)
Supplement: Additional file 1 — ProteinShader program without source code. This compressed file contains the complete ProteinShader program including associated libraries, but no source code. A README.txt file gives an overview of the ProteinShader distribution, and the index.html file in the help subdirectory has directions on getting started with the program as well as a set of tutorials. [file 1472-6807-9-19-S1.zip › ProteinShader-beta-0_9_4-binary/help/api/org/proteinshader/graphics/typography/GlyphDemo.html]

GlyphDemo (ProteinShader API)


|  |  |  |
| --- | --- | --- |
| **Overview** \| **Package** \| **Class** \| **Use** \| **Tree** \| **Deprecated** \| **Index** \| **Help** | |  |
| **PREV CLASS**   **NEXT CLASS** | **FRAMES**    **NO FRAMES**     **All Classes** |  |
| SUMMARY: NESTED \| FIELD \| CONSTR \| METHOD | DETAIL: FIELD \| CONSTR \| METHOD |  |


---


## org.proteinshader.graphics.typography Class GlyphDemo

```
java.lang.Object
  org.proteinshader.graphics.typography.GlyphDemo
```

---

``` public class GlyphDemo extends Object ```

Provides a driver to perform some basic tests on classes in the
typography package.

---

**Constructor Summary**

`GlyphDemo()`


| **Method Summary** | |
| --- | --- |
| `static void` | `main(String[] args)`             Creates an instance of TextLabelFactory and has it read in the default set of glyphs. |
| `static void` | `printTextLabel(TextLabel label)`             Prints information from the TextLabel, including the bitmap. |

**Methods inherited from class java.lang.Object**

`clone, equals, finalize, getClass, hashCode, notify, notifyAll, toString, wait, wait, wait`

**Constructor Detail**

### GlyphDemo

```
public GlyphDemo()
```


**Method Detail**

### main

```
public static void main(String[] args)
```

:   Creates an instance of TextLabelFactory and has it read in the
    default set of glyphs.

---


### printTextLabel

```
public static void printTextLabel(TextLabel label)
```

:   Prints information from the TextLabel, including the bitmap.

    :   **Parameters:**: `label` - the text label to print info on.


---


|  |  |  |
| --- | --- | --- |
| **Overview** \| **Package** \| **Class** \| **Use** \| **Tree** \| **Deprecated** \| **Index** \| **Help** | |  |
| **PREV CLASS**   **NEXT CLASS** | **FRAMES**    **NO FRAMES**     **All Classes** |  |
| SUMMARY: NESTED \| FIELD \| CONSTR \| METHOD | DETAIL: FIELD \| CONSTR \| METHOD |  |


---

# *Copyright © 2007-2008*
